# Structural cause of dysphagia detected during videofluoroscopic swallow study

**DOI:** 10.1002/ccr3.1057

**Published:** 2017-07-07

**Authors:** Ezekiel Wong Toh Yoon, Syu Kabuto

**Affiliations:** ^1^ Department of Internal Medicine (Gastroenterology) Hiroshima Kyoritsu Hospital Hiroshima Japan; ^2^ Department of Palliative Care Hiroshima Kyoritsu Hospital Hiroshima Japan

**Keywords:** Aspiration pneumonia, dysphagia, esophageal cancer

## Abstract

Dysphagia can be caused by many different underlying conditions. The assessment and management of dysphagia depend on each individual patient, often requiring a multidisciplinary approach. Structural cause of dysphagia can be dealt with using endoscopic interventions before the patient's general status deteriorates.

A 72‐year‐old man was referred to receive percutaneous endoscopic gastrostomy (PEG). About 2 months ago, he was treated in a tertiary hospital for aspiration pneumonia but dysphagia persisted and there was difficulty in replacing his nasogastric tube. During routine videofluoroscopic swallow study conducted by our dysphagia team, severe aspiration was observed as the ingested material consistently regurgitated from the upper esophagus. A chest radiograph was taken immediately after the study (Fig. 1).

**Figure 1:**
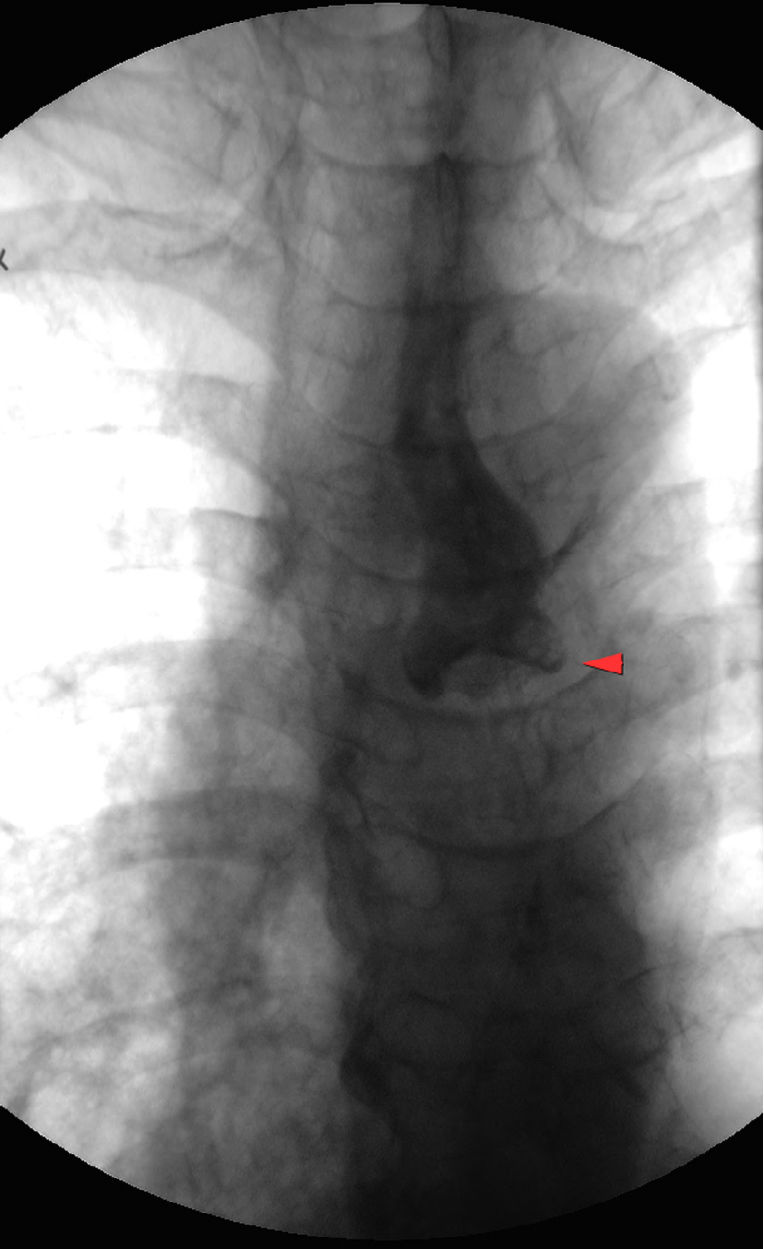
Anterior chest radiograph taken after videofluoroscopic swallowing study (red arrow head pointing to the pooling of contrast material above the mid‐thoracic esophagus).

## What is Your Diagnosis?

**Figure 2:**
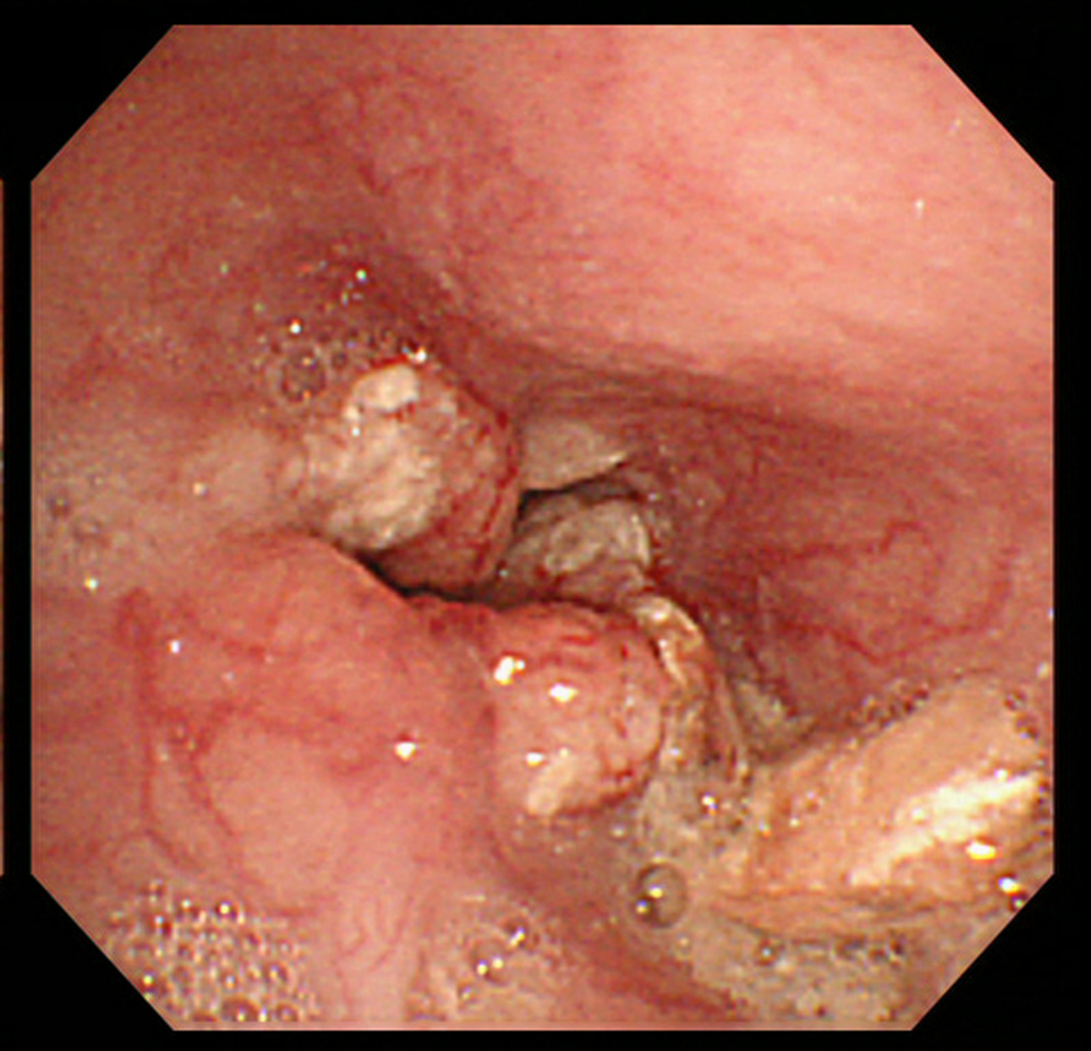
Endoscopic image revealing severe stenosis of the mid‐thoracic esophagus due to esophageal cancer.

**Figure 3:**
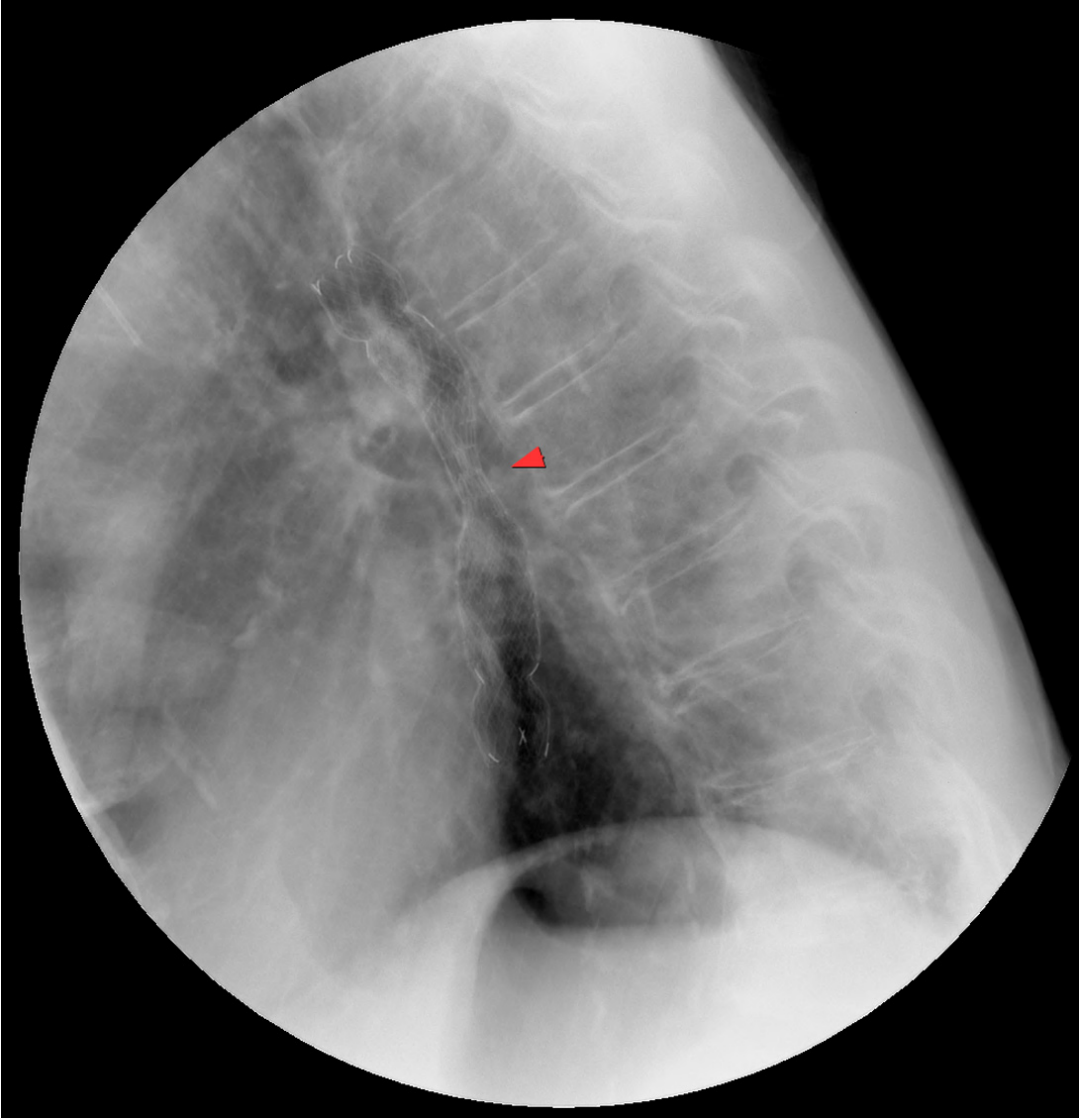
Lateral chest radiograph after self‐expandable metallic stent insertion (red arrow head pointing to the area of stenosis).

Upper gastrointestinal endoscopy was recommended and confirmed severe stenosis of the mid‐thoracic esophagus from esophageal cancer (Fig. 2). Esophageal stenting alleviated some of his symptoms (Fig. 3), but due to his poor general status, curative surgery was deemed not suitable and he was transferred to palliative care. Although PEG followed by swallowing therapy can be effective in some patients with dysphagia 1, the underlying cause for each patient needs individual assessment and management 2. A multidisciplinary approach may be useful to rule out structural causes, and noninvasive interventions, such as in this case, may prevent the deterioration of general status if performed earlier.

## Authorship

EWTY: prepared the manuscript. SK: had an advisory role in the management of the patient.

## Conflict of Interest

None declared.
